# Determining the association between fibromyalgia, the gut microbiome and its biomarkers: A systematic review

**DOI:** 10.1186/s12891-020-03201-9

**Published:** 2020-03-20

**Authors:** Sharon Erdrich, Jason A. Hawrelak, Stephen P. Myers, Joanna E. Harnett

**Affiliations:** 1 Faculty of Medicine and Health, School of Pharmacy, The University of Sydney, Sydney, New South Wales Australia; 2 College of Health and Medicine, University of Tasmania, Hobart, Tasmania Australia; 3 NatMed Research Unit, Division of Research, Southern Cross University, Lismore, New South Wales Australia

**Keywords:** Fibromyalgia, Fibromyalgia syndrome, Gastrointestinal microbiome, Biomarkers, Systematic review

## Abstract

**Background:**

The association between fibromyalgia and irritable bowel syndrome is well-established. Alterations in the composition and diversity of the gut microbiome in irritable bowel syndrome have been reported, however, this association is poorly understood in fibromyalgia.

Our aim was to summarise the research reporting on the gastrointestinal microbiome and its biomarkers in people with fibromyalgia.

**Methods:**

A systematic review of published original research reporting on the gastrointestinal microbiota and its biomarkers in adults with a diagnosis of fibromyalgia was undertaken.

**Results:**

From 4771 studies, 11 met our inclusion criteria and were separated into four main groups: papers reporting *Helicobacter pylori*; other gut bacterial markers; metabolomics and other biomarkers, which included intestinal permeability and small intestinal bacterial overgrowth.

**Conclusion:**

The results suggest there is a paucity of quality research in this area, with indications that the gut microbiota may play a role in fibromyalgia within the emerging field of the gut-musculoskeletal axis. Further investigations into the relationship between the gut microbiota, gut dysfunction and fibromyalgia are warranted.

## Background

It has been almost half-century since the term fibromyalgia replaced fibrositis [1], yet the condition remains idiopathic, poorly understood and difficult to treat [2]. Its cardinal symptom is pain, which must be both widespread and chronic to meet the current diagnostic criteria.

The prevalence of fibromyalgia shows large variability. A review of 39 studies conducted across 19 countries indicates prevalence ranges from 0.2 to 6.6% [3]. The associated morbidity is not insignificant and the costs, both direct and indirect, contribute to a significant socioeconomic burden [4].

Without laboratory evaluations that contribute to the diagnosis of the condition, it is also difficult to diagnose [5]. Therefore, clinical presentation of the typical pain and the presence of associated somatic and psychological symptoms form the basis of current diagnosis.

To date the pathophysiology of fibromyalgia remains elusive. No causative agent has been identified, however, a role for a range of different infections has been suggested [6]. Nervous system dysfunction is implicated and neurotransmitters are the targets of a number of drugs approved for fibromyalgia [7]. Recently, Albrecht and colleagues have suggested a possible role for microglial activation and neuroinflammation [8]. Pathological changes in epidermal nerve fibres has also been implicated, with evidence for the association nearing 50% [9]. The role of oxidative stress, autoimmune markers, proteomics, genetics and a range of hormones have all been implicated with inconsistent results [10]. Inflammation has never been clearly identified. A systematic review and meta-analysis of 13 studies reporting inflammatory biomarkers in fibromyalgia found only plasma interleukin-6 (IL-6) to be higher in patients, compared to controls.

Fibromyalgia is associated with a range of somatic symptoms and gastrointestinal disorders. Our group have recently conducted a systematic review of the co-morbidity of FGID in fibromyalgia and found this area to be under-explored, with the majority of studies focusing on irritable bowel syndrome (IBS) [11]. IBS has been associated with alterations in the composition and diversity of the gastrointestinal microbiota [12]. Individuals with both fibromyalgia and IBS report higher symptom severity and poorer quality of life [13, 14]. Interest in the role of the gastrointestinal microbiome on both the gut-brain axis and systemic disorders has increased exponentially in recent years and a comprehensive review of this topic as it relates to fibromyalgia is warranted.

The aim of this systematic review was to examine and summarise the research reporting on the composition and diversity of the gut microbiome and associated biomarkers in adults with fibromyalgia.

## Methods

This review was registered with PROSPERO (https://www.crd.york.ac.uk/PROSPERO/). [registration number pending].

### Data sources

A search of Medline, CINAHL, Embase and Web of Science databases was made using the terms in Table 1, conducted over 06–08 August 2019. Results were exported into EndNote X9.2 (Thomson Reuters Inc., Philadelphia, PA, USA.) and duplicate records deleted.

**Table 1:** Search used for the Medline database to identify literature reporting on fibromyalgia and terms related to gut microbiome and/or biomarkers of gut bacteria

Search	Search term(s)
1.	fibromyalgia.mp. or Fibromyalgia/ or fibromyositis or fibrositis or "muscular rheumatism"
2.	"c-reactive protein.mp. or C-Reactive Protein/ or CRP.mp. or interleukin.mp. or exp. Interleukins/ or IL-1B.mp. or IL-6.mp. or IL-10.mp. or IL-17.mp. or cytokine* or cytokines).mp. or exp. Cytokines/ or chemokine.mp. or tumor necrosis factor.mp. or exp. Tumor Necrosis Factor-alpha/ or TNF*.mp. or exp. Creatine Kinase/ or ("creatine kinase" or protease).mp. or ((metabolic or lactic) and acidosis).mp. or acidosis/ or exp. acidosis, lactic/ or anion gap.mp. or Acid-Base Equilibrium/
3.	((lactulose adj3 mannitol) or (lactulose and mannitol)).mp. or (zonulin or occludin).mp. or Occludin/ or citrulline.mp. or Citrulline/ or zonula occluden*.mp. or lipopolysaccharide*.mp. or exp. Lipopolysaccharides/ or LPS.mp. or flagellin.mp. or (endox* or (bacter* adj3 toxin*)).mp. or (histamine or tryptase).mp. or (calprotectin or eosinophil protein x).mp. or (secretory IgA or sIgA).mp. or *Immunoglobulin A, Secretory/ or (((butyric or acetic or propionic or lactic) and acid) or (butyrate or acetate or propionate or lactate)).mp. or exp. Fatty Acids, Volatile/ or short chain fatty acid*.mp. or SCFA.mp. or (trimethylamine N-oxide or TMAO).mp. or Ammonia/ or (ammonia or hippuric or 2-hydroxyisobutyric).mp. or (creatine or succinic or taurine or serine or arginine or glutam*).mp. or Methane/ or methane.mp. or Hydrogen/ or hydrogen.mp. or "hydrogen sul?ide".mp. or exp. Biomarkers/ or biomarker*.mp. or metabolite*.mp. or metabolome.mp. or exp. Metabolome
4.	2 or 3
5	(((gastrointestin* or gut) adj3 microbio*) or (intestin* adj3 flora) or (intestin* adj3 bacteria)).mp. or exp. gastrointestinal microbiome/ or gastrointestinal microbiome.mp. or microbio*.mp. or exp. Microbiota/ or exp. Mycobiome/
6.	Lactobacillus/ or lactobacill*.mp. or Bifidobacterium/ or bifidobacter*.mp. or exp. Probiotics/ or probiotic*.mp. or (akkermansia or f?calibacter*).mp. or (clostrid* or bilophil*).mp. or Bilophila/ or Desulfovibrio/ or desul?ovibrio.mp. or methano*.mp. or exp. Methanobrevibacter/ or (proteobacter or citrobacter or actinobacter*).mp. or (Roseburia or Eubacteri* or Subdoligranulum or Anaerostipes or Lachnospiraceae).mp. or exp. Eubacteria/ or exp. Prevotella/ or prevotella.mp. or klebsiella.mp. or exp. Klebsiella/ or collinsella.mp. or veillonella.mp. or Veillonella/ or exp. Bacteroidetes/ or bacteroid*.mp. or firmicutes.mp. or Firmicutes/
7.	exp Gram-Positive Bacteria/ or exp. Gram-Negative Bacteria/ or ((gram-negative or gram-positive) and bacteria).mp. or exp. aerobic bacteria/ or (aerobic adj3 bacter*).mp. or exp. anaerobic bacteria/ or (anaerobic adj3 bacter*).mp. or helicobacter pylori.mp. or Helicobacter pylori/ or "*H. pylori*".mp. or exp. Candida/ or candida.mp. or exp. Saccharomyces/ or saccharomyces.mp. or rhodotorula.mp. or Rhodotorula/ or yeast.mp.
8.	5 or 6 or 7
9.	4 or 8
10.	1 and 9
11.	Limit 10 to English language, Humans, Adults
12.	Limit 11 to publication date: 01 January 1976^a^ to present

^a^the year "fibromyalgia" became the official term for the condition 1. Inanici F, Yunus MB: History of fibromyalgia: past to present. *Current Pain and Headache Reports* 2004, 8(5):369–378

### Study selection

Further screening to remove conference abstracts, studies involving children (< 18 years of age), animals, reviews, case reports or papers irrelevant to our objectives. Full-text articles were retrieved and linked to the corresponding EndNote record. Reference lists of included articles were reviewed for other studies relevant to our objectives. Articles were scanned for diagnostic criteria used; those not specifying accepted guidelines for diagnosis were excluded. We also excluded papers reporting systemic biomarkers not evaluated in the context of gastrointestinal symptoms or gut flora.

**Fig. 1:**
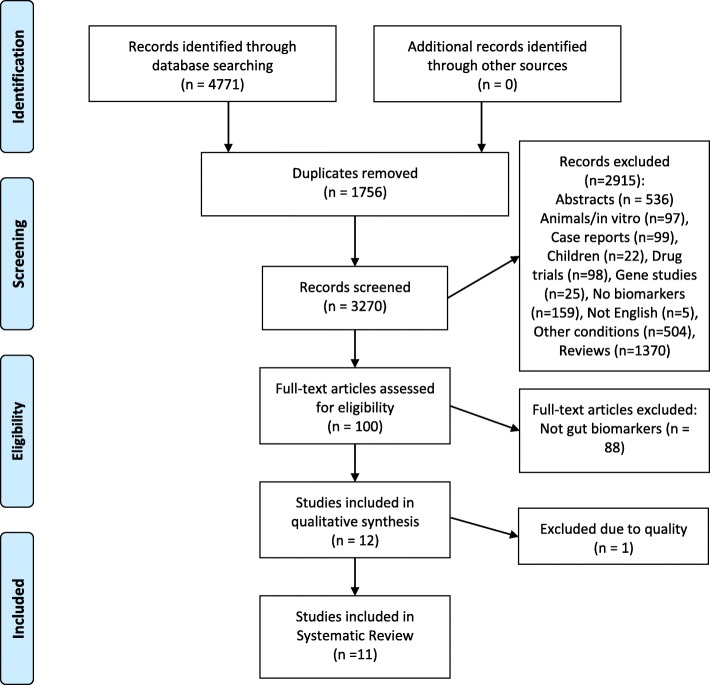
PRISMA Flow Diagram Showing Study Selection Process

The selection process is presented in Fig. 1, utilising the Preferred Reporting Items for Systematic Reviews and Meta-Analyses (PRISMA) flow diagram [15].

### Data extraction

The following data was extracted to a Microsoft® Excel® spreadsheet: lead author, year, country of publication, gender and age of cohort, diagnostic criteria used for fibromyalgia, bacteria or biomarker thereof reported, summary of results.

### Quality assessment

All studies were reviewed by two authors (SE and JEH) who screened for inclusion criteria and conducted a quality assessment using the Joanna Briggs critical appraisal tool “Checklist for case-control studies” [16], with additional quality criterion including: diagnostic criteria used to identify fibromyalgia, the description of cohort/s, and evidence that ethics/consent had been granted, giving a total of 16 criteria for evaluation. Scores were then converted to percentages for reporting.

## Results

A total of 4771 articles were retrieved. The PRISMA flow diagram (Fig. 1) shows the selection process and number of studies included and excluded at each step to arrive at the final eleven studies, conducted in 7 countries, included in this analysis. There was a high degree of heterogeneity across the studies, which were of variable quality (Table 2).

**Table 2:** Characteristics of 11 papers investigating biomarkers related to the gastrointestinal microbiome in subjects with fibromyalgia

Author (Year)	Country	Cohort		Study Type	FMS criteria	Controls n, Type	Biomarker/s	Quality Assessment %
		N (% female)	Age					
Pimentel (2004) [17]	USA	42 (86)	46.6 ± 0.3	Case-control	ACR 1990	22 IBS	SIBO	53.1
Michalsen (2005) [18]	Germany	35 (91)	52.0 ± 10.0 51.6 ± 13.3^a^	Case-control	ACR 1990	16 RA	Stool culture Stool pH sIgA	56.3
Goebel (2008) [19]	Germany	40^c^ (80)	48 ± 11	Case-control	ACR 1990	57 HC 17 CRPS	*H. pylori*Bacteria (serum antibodies)Intestinal permeability	75.0
Akkaya (2011) [20]	Turkey	65 (100)	36.21 ± 7.42	Case-control	ACR 1990	41 HC	*H. pylori*	75.0
Olama (2013) [21]	Egypt	100 (100)	33.2 ± 4.36	Case-control	ACR 1990	100 HC	*H. pylori*	81.3
Rodrigo (2013) [22]	Spain	104 (89)	50 ± 8^b^	Case-control	ACR 1990	125 IBS	*H. pylori*	75.0
Gezici (2014) [23]	Turkey	32 (88)	38.5 ± 8.6	Prospective	ACR 1990	–	*H. pylori*	43.8
Malatji (2017) [24]	South Africa	18 (100)	45.5	Case-control	ACR 1990	11 FC 10 MC 41 YC	Metabolomics	75.0
Malatji (2019) [25]	South Africa	17 (100)	45.5 (ns)	Case-control	ACR 1990	11 FC 10 MC 41 YC	Metabolomics	75.0
Clos-Garcia (2019) [26]	Spain	105 (70)	52.52 ± 10.3	Case-control	ACR 1990	54 HC	Microbiome Metabolomics	81.3
Minerbi (2019) [27]	Canada	77 (100)	46 ± 8	Case-control	ACR 2016	11 FC 20 HM 48 UC	Microbiome Metabolites	87.5

Key: *ACR* American College of Rheumatology; *FMS* Fibromyalgia; *JB* Joanna Briggs; *ns* not stated; *IBS* irritable bowel syndrome; *SIBO* small intestinal bacterial overgrowth; *RA* rheumatoid arthritis; *HC* healthy controls; *CRPS* chronic regional pain syndrome; *FC* family controls; *MC* matched controls; *YC* young controls; *HM* household members; *UC* unrelated controls; ^a^ = age (SD) of FMS in group B; ^b^ = age of IBS cohort 51 ± 8; ^c^ = 40 FMS patients, serology data for 33.

A total of 618 subjects with fibromyalgia (90% female [*n* = 554]), and 635 controls, of whom 180 (29%) had other health conditions were included. Two papers presented different data from the same cohort which is included once in the population totals.

Eight papers evaluated markers for specific gut microbes: five reported on *Helicobacter pylori* [19–23], and are reported as a group. Four described biomarkers of the gut microbiota [18, 19, 26, 27]; four explored metagenomics/metabolites or metabolomics [24–27], and three [17–19] reported other biomarkers, such as intestinal permeability. Four studies [18, 19, 26, 27] examined more than one type of biomarker and so are included in each of the appropriate sections below.

A summary of the key information of the 11 included articles is presented in Table 2.

### Helicobacter pylori

Table 3 details results of five studies evaluating *H. pylori* in patients with fibromyalgia.

**Table 3:** Results from 5 Studies Investigating H. pylori

Author (Year)	Cohort	Material ***H. pylori*** marker	***H. pylori*** positive (%)		
			FMS	Controls	***p***- value
Goebel (2008) [19]	33 FMS 17 CRPS 57 HC	Serum antibodies IgA or IgG	33.0	HC: NT CRPS: 47.0	n.s.
Akkaya (2011) [20]	65 FMS 41 HC	Serum antibodies IgA IgG	30.8 67.7	17.1 43.9	0.169 0.025
Olama (2013) [21]	100 FMS 100 HC	Serum antibodies IgA IgG	50.0 68.0	24.0 32.0	< 0.001 < 0.001
Rodrigo (2013) [22]	104 FMS 125 IBS	Gastroduodenal biopsyH&E + Giemsa staining, polyclonal anti-*H. pylori* antibody, microbial cultures	42.0	46.0	n.s.
Gezici (2014) [23]	41^#^ FMS	Gastric biopsy Culture	78.0	–	–

Key: ^#^ all had dyspepsia; *FMS* fibromyalgia; *HC* healthy controls; *CRPS* chronic regional pain syndrome; *IBS* irritable bowel syndrome; *NT* not tested; *NA* not reported; *n.s.* not statistically significant.

Biopsy results from Rodrigo’s study also revealed significantly higher rates of intraepithelial lymphocytosis (IEL) (Marsh stage 1) and villous atrophy (Marsh stage 3) in the group with fibromyalgia plus IBS, compared to the IBS-only group (56% vs 16 and 7% vs 2%, respectively), (both *p* < 0.001). As a result of this investigation, seven of the 104 patients with fibromyalgia were subsequently diagnosed with coeliac disease [22].

### Gastrointestinal microbiota and antibodies

Four studies are included in this group, as presented in Table 4. In addition to serum antibodies to two gut-specific organisms, Goebel et al., [19] assessed results against measures of intestinal permeability (IP) (see Table 6). Three studies evaluated the microbial composition of faecal specimens, however, all employed different methodology.

**Table 4:** Results from 4 studies Investigating Gastrointestinal Microbiota Taxa and Antibodies

Author (Year)	Patients Controls	Material & Procedure	Results	***p-***value	Other
Michalsen (2005) [18]	21 FMS 9 RA	Faeces Culture	No differences	n.s	pre- vs post-fasting
	14 FMS 7 RA	Faeces Culture	No differences	n.s	pre- vs post-MedDiet
Goebel (2008) [19]	33 FMS17 CRPS 57 HC	Serum antibodies *Yersinia***or***Campylobacter*	FMS 27% positive CRPS 13% HC Not tested	NR	Trend for seropositivity assoc. with IP (Table 4)
Minerbi (2019) [27]	77 FMS 11 FC 20 HM 48 UC	Faeces 16S rRNA gene (V5-V6 region) / metagenome	*↓ Faecalibacterium* and *Bacteroides, ↑ Intestinimonas, Flavonifractor, Butyricoccus Eisenbergiella* and *Enterobacter.*	< 0.01	Non-significant differences in sample diversity Variance FMS vs UC
Clos-Garcia (2019) [26]	105 FMS 54 HC	Faeces 16S rDNA microbiome(V3–V4 region)	FMS: Absent families: Bifidobacteriaceae and Bacteroidales*↑ Dorea, Roseburia, Alistipes, Papillibacter, Subdoligranulum↓ Bacteroides, Bifidobacterium, Eubacterium, Clostridium*	All *p* ≤ 0.05	Controls had higher diversity

Key: *n.s*. not statistically significant; *NR* not reported; *FMS* Fibromyalgia; *RA* rheumatoid arthritis; *HC* healthy controls; *CRPS* chronic regional pain syndrome; *IP* intestinal permeability; *FC* family controls; *HM* household members; *UC* unrelated controls.

### Metabolomics/metabolites

The urinary microbial metabolism data reported in two of the studies included in this review were obtained from the same cohort [24, 25]. Two other studies evaluated serum markers for a range of metabolites, proteins and gene expression [26, 27], and three compared patients with more than one type of control [24, 25, 27]. These are summarised in Table 5.

**Table 5:** Results from 3 studies (4 papers) Investigating Metabolites and Metabolomics

Author (Year)	Subjects Controls	Material Biomarker	FMS Subjects	***P*** value for diff
Malatji (2017) [24]	18 FMS11 FC10 MC41 YC	UrineNMR metabolome	Compared to YC ↑ hippuric acid ↑ succinic acid ↑ lactic acid ↑ 2-Hydroxyisobutyric acid	*p* = 0.0966(ns) *p* = 0.0001 (MW) *p* = 0.0044 *p* = 0.0001
Malatji (2019) [25]	17 FMS11 FC10 MC41 YC	UrineGC-MS metabolome	↑ 14 metabolites c.f. MC↑ 4-hydroxyisobutyric acid c.f. MC & YC ↑ arabinose c.f. MC	All < 0.001 (MW & BF) *p* = 0.00109
Minerbi (2019) [27]	77 FMS11 FC20 HM48 UC	Serum Metabolites	Compared to UC: ↑serum butyrate ↓serum propionic acid ↓isobutyric acid	*p* = 0.005*p* = 0.006 *p* = 0.056 (ns)
Clos-Garcia (2019) [26]	105 FMS54 HC	Serum Metabolome	↓ gadC, glnA, glsA2, gadB1, gadB2↑ lysine, ornithine ↑threonine, homoserine, glutamine, and arginine^a^	*p* < 0.05 *p* < 0.05 *p* < 0.001

Key: *FMS* Fibromyalgia; *NMR* nuclear magnetic resonance spectrometry; *GC-MS* gas chromatography–mass spectrometry: *HC* healthy controls; *FC* family controls; *MC* matched controls; *YC* young controls; *HM* household members; *UC* unrelated controls; *c.f.* compared to; *MW* Mann Whitney test; *BF* Bonferroni-Holm test; *ns* not significant; *gad* glutamate decarboxylase; *gln* glutamine synthetase; *gls* glutaminase: ^a^ all identified as likely of bacterial origin.

Alterations in levels of urine metabolites reported in the first of Malatji’s papers [24] were predicted to be associated with gut bacteria. Employing a more targeted approach, the same team later reported the presence of the monosaccharides sorbose, rhamnose and tagatose which are typically not found in human urine [25]. One mechanism proposed by the authors for their presence was bacterial degradation of plant-derived carbohydrates.

Clos-Garcia et al. [26] also concluded that some of the altered metabolites, as well as both up- and down-stream products, may be bacterial in origin.

### Other biomarkers

Three studies [17–19] outlined in Table 6, evaluated markers not included in the groups above. In Michalsen’s study [18], fibromyalgia and rheumatoid arthritis patients were allocated into two separate arms, each of which undertook a different dietary intervention (either a Mediterranean diet or fasting).

**Table 6:** Results From 3 Studies Investigating Other Gut-Related Markers

Author (Year)	Cohort	Material / Biomarker	Results		***p***-value for diff
			FMS	Controls	
Pimentel (2004) [17]	42 FMS^a^ 111 IBS 15 HC	Breath(SIBO)	100%	IBS 84% HC 20%	< 0.05 < 0.0001
Michalsen (2005) [18]	21 FMS9 RA	StoolpH & sIgAfollowing fasting		No differences between FMS vs RA groups or with different interventions	n.s.
	14 FMS7 RA	StoolpH & sIgAfollowing MedDiet			n.s.
Goebel (2008) [19]	40 FMS 17 CRPS 57 HC	UrineGD permeabilitySI permeability	32.5%37.5%	CRPS 35.3%, HC 5%CRPS 17.6%, HC 0%	< 0.0001^b^ < 0.0002^&^

Key: ^a^ = 22 (54%) had IBS; ^b^ = *p*-value compared to controls; *FMS* Fibromyalgia; *CRPS* chronic regional pain syndrome; *HC* healthy controls; *GD* gastroduodenal; *SI* small intestine; *IBS* irritable bowel syndrome; *RA* rheumatoid arthritis; *MedDiet* Mediterranean Diet; *SIBO* small intestinal bacterial overgrowth; *n.s.* not significant.

Pimentel et al. [17] investigated the prevalence of small intestinal bacterial overgrowth (SIBO) in patients with fibromyalgia, IBS and normal controls. Both peak and area-under-the-curve (AUC) values for hydrogen production where significantly higher in those with fibromyalgia compared to controls both with and without IBS.

Both small intestinal and gastroduodenal permeability was evaluated in Goebel’s study [19] employing lactulose-mannitol recovery testing. Follow-up data collected in the week after testing, while not available for all participants, revealed 2 days of moderate-to-severe diarrhoea in 13 of the 31 subjects.

### Gut biomarkers and interventions associated with fibromyalgia symptoms

Studies exploring the relationship between *H. pylori* and fibromyalgia were conflicting. Akkaya’s study found no differences in regards to any of the clinical features evaluated [20], whereas Olama et al., described significant associations between *H. pylori* immunoglobulin-G (IgG) -positive patients and disease markers, including: post-exertion pain, morning stiffness, confusion, mood, tension headache, sleep disturbance, dyscognition, changes in appetite and fatigue (all *p* < 0.05) [21]. Gezici’s group reported a significant reduction in pain, as evaluated by the number of tender points, following *H. pylori* eradication (*p* < 0.001) [23].

A significant association between the differential abundance of several taxa and some indices of the 2016 ACR Fibromyalgia criteria (such as pain intensity, widespread pain index (WPI), dyscognition), and quality of life scores (including fatigue) was reported by Minerbi’s group (all *p* < 0.05). Some operational taxonomic units were found to be inversely related to many of the same indicators in unrelated controls [27]. Genus-level composition of the gut microbiome weakly correlated to pain indicators in Clos-Garcia’s report, with the strongest correlations in micro ribonucleic acid (miRNA) data, followed by serum proteins and metabolomics [26]. Malatji et al. (2017) described specific combinations of metabolites (creatine & succinic acid +/− taurine) relating to pain scores and loss of energy [24]. In spite of indicating that 88% of their small cohort had some degree of gastrointestinal dysfunction, possibly classified as IBS, details were not described; nor was any attempt made to relate digestive symptoms to the measured metabolites [25].

Pain scores correlated significantly with both peak and AUC hydrogen gas production (r = 0.42, *p* < 0.01 and r = 0.37, *p* < 0.05) on breath testing by Pimentel’s group [17]. Treatment of SIBO resulted in significant reductions in pain (*p* < 0.05). A non-significant improvement in clinical measures was described in those patients following the fasting regime in Michalsen’s study [18], and Goebel reported no association between IP and the pain of either chronic regional pain syndrome (CRPS) or fibromyalgia [19].

## Discussion

We set out to systematically evaluate the literature reporting data associated with the gut microbiota in people with fibromyalgia. The review identified five broad areas of research, but to date no single gut-fibromyalgia biomarker has been validated. Some studies demonstrated variability in markers associated with the gut microbiota in patients with fibromyalgia compared to healthy controls [24].

A wide range of research methods were detected and lack of control for factors which may impact the biomarkers being investigated was common. This included failure to measure dietary patterns, smoking and drug use. Importantly, of the included studies that evaluated the composition of the gut microbiome, the methods used were different, making comparisons problematic. Some statistically significant differences were reported, but due to the high degree of variability in both the methods and markers evaluated, and the presence of important confounding factors, it is difficult to draw any strong conclusions.

### Helicobacter pylori

While several papers reported a relationship between *H. pylori* and fibromyalgia the results are inconsistent. This is probably due to a combination of different identification methods and potential confounders, such as the retention of patients in the control group with CRPS who met fibromyalgia criteria [19].

None of the included studies utilised stool antigen or 13C-urea breath tests, and two [22, 23] performed culture following biopsy. Each of these are more reliable than serology for *H. pylori* diagnosis [28].

Rodrigo et al., [22] described *H. pylori* at similar rates in patients with IBS regardless of fibromyalgia status. They also found coeliac disease (CD) in fibromyalgic patients at seven times the population rate and more than double that reported in fibromyalgia elsewhere [29]. A 2012 study of 376 CD patients demonstrated 22% met the 1990 ACR criteria for fibromyalgia [30] suggesting a bidirectional relationship. In addition to its association with CD and non-coeliac gluten sensitivity [31], IEL has also been observed in *H. pylori* infections [32], although other work has refuted this relationship [33]. No other study included in this review evaluated subjects for CD and neither CD nor IEL were in our search terms.

All subjects in Gezici’s Turkish study had fibromyalgia and dyspepsia; 78% were positive for *H. pylori* [23], similar to the overall prevalence in that country [34]. Cytotoxin-associated gene A (CagA)-positive strains of *H. pylori* are associated with a higher risk of dyspepsia [35], but none of the studies included in this review evaluated virulence factors.

In summary, the studies provide weak evidence that *H. pylori* may play a role in the symptomology of fibromyalgia, and while the exact relationship is uncertain, the improvement seen in pain scores just 3 weeks after antibiotic treatment of *H. pylori* [23] warrants a systematic and rigourous investigation.

### Gut microbiota

With rapid developments in microbiome technology over the last two decades, it is not surprising our review identified a diverse set of methodological approaches employed in the assessment of antimicrobial communities. In 2005, bacterial culture was the accepted procedure as DNA sequencing was in its infancy.

Michalsen’s study [18] demonstrated no differences in the microbiota, however, this finding is limited by an absence of a healthy control group. Additionally, fibromyalgia and rheumatoid arthritis (RA) commonly coexist [36], and it is unclear whether participants with fibromyalgia were screened for RA.

A higher prevalence of positive antibodies to either *Yersinia enterocolitica* or *Campylobacter jejuni* (data not separated) in subjects with fibromyalgia compared to those with CRPS is difficult to interpret [19]. While these organisms commonly coexist, double-positivity for *H. pylori* plus *C. jejuni* tends to be low [37] and *H.pylori*, which may be protective against infection-induced diarrhoeal disease [38], was high in this cohort. Other factors, including use of *H. pylori* serology, absent data for healthy controls, and seasonal variability of *C. jejuni* infection [37, 39], create further limitations to both the interpretation and generalisability of these findings.

Imbalances in several genera were evident in the gut microbiota data reported by both Minerbi and Clos-Garcia [26, 27] (Table 4). Differences in sample collection protocols, the regions of amplification and the sequencing platforms makes comparability difficult.

In addition to assessing the microbiome, Minerbi et al. [27] evaluated relationships with a range of other variables, including age, diet, drugs and physical activity. A particular strength of the study was the inclusion of genetic (first-degree relatives), environmental (household cohabitants) and normal (non-related, not cohabiting) controls and a dietary assessment for comparison between groups.

Several significant (*p* < 0.05), positive correlations were reported between fibromyalgia symptoms and some taxa. Differences in the microbiota were associated with the diagnosis and symptoms of fibromyalgia. Of note, *Bacteroides spp.* was positively correlated with total symptom score on the Fibromyalgia Impact Questionnaire (FIQ) and the 2016 ACR diagnostic criteria. Increased abundance of *Parabacteroides merdae* and a non-significant increase in *Akkermansia muciniphila* was also reported. These species appear to function synergistically, resulting in lower faecal gamma-glutamylation activity, with a subsequent increase in hippocampal gamma-Aminobutyric acid (GABA): glutamate [40]. While the relevance of this in fibromyalgia is unclear, and an in depth discussion is outside the scope of this review, it is interesting that glutamate, which functions as an excitatory neurotransmitter and plays a role in pain signaling [41, 42] has been reported at higher levels in the insula [43] and in the interstitial compartment of muscle of fibromyalgics [44]. Altered brain levels of glutamate + glutamine have been associated with increased pain sensitivity [45] and other research, identified lower levels of the neurotransmitter GABA in the same area of the brain [46].

Clos-Garcia et al. [26] used quantitative polymerase chain reaction (qPCR) to determine bacterial gene expression related to glutamate metabolism and despite finding five out of six glutamate degrading genes at significantly higher levels in fibromyalgics than controls (which would be expected to result in less glutamate), higher serum levels of glutamate were found in the fibromyalgia group. While this appears contradictory, the higher serum glutamate is consistent with other data reporting a role for glutamate in the central nervous system (CNS) [47] and in pain signaling [41]. The bacteria involved in glutamate and GABA metabolism in these two studies were different: *Bifidobacterium* and *Lactobacillus* in Clos-Garcia’s study [26], and *Akkermansia muciniphila* and *Parabacteroides spp.*, in Minerbi’s [27]. These differences are likely to be attributable to the region of the genome sequenced. Clos-Garcia’s group also described a “discrete” lower bacterial diversity in their patients, and higher abundance of several butyrate producers, congruent with findings of higher levels of the butyrate in the serum.

A high rate of drug use, both prescription and complementary medicines, was reported in Clos-Garcia’s study. Data provided in their Supplemental Table 1, detailed 169 agents, including several with psychotropic and CNS effects. Antidepressants, benzodiazepines, antiepileptics and gabapentin and were used by 76, 66, 28 and 10% of patients respectively and 23% were current smokers. Minerbi’s group [27] also collected information regarding drug use and smoking status, but little attention was given to these as confounders, nor was detail regarding the type, or extent of drug use or smoking provided. Drugs of many kinds may impact gut bacteria and their metabolism, and vice-versa: smoking is associated with an altered gut microbial profile [48] and the *Ruminococcus* genus (which was overexpressed in Clos-Garcia’s patient group) can impact antidepressant metabolism, drugs which also alter the gut microbiota [49].

The understanding of the wide-reaching role of gut bacteria in a plethora of metabolic processes and health conditions has advanced significantly in recent years. The advent of deep sequencing of the microbiome has enabled a new era of discovery which is rapidly evolving. Due to the limited number of studies evaluating the composition of the gastrointestinal microbiota in our results, the heterogeneity of techniques used, variance in findings, and issues around drug use, it is difficult to draw any firm conclusions regarding microbiome alterations in this patient population. This Pandora’s box is of intricate complexity [50], such that meaningful results would possibly be best obtained by studying these factors in a much larger, drug-naïve cohort. To date, no extensive analysis has been undertaken to simultaneously evaluate the composition of the gastrointestinal tract microbiota, its metabolites, luminal neurotransmitters and brain neurochemistry in people with fibromyalgia. Future studies should consider such analyses.

### Metabolomics

Several variations in metabolomic markers suggest altered microbial metabolism, but these are not consistent across the studies. The previously identified problems regarding the impact of drug intake and different dietary patterns in the patient populations confounds results.

Alterations in levels of short chain fatty acids (SCFA) suggest a role for altered bacterial populations, though few, and at times conflicting, associations were reported. Higher serum butyrate was reported by Minerbi, which is consistent with increased urinary butyrate seen in Malatji’s study but is at odds with the lower expression of butyrate-producers reported by both Minerbi and Clos-Garcia. Interestingly, lower levels of butyrate-producing bacteria are reported in IBS [51], which may be a reflection of its roles in regulating the colonic mucosa, intestinal motility and modulating visceral hypersensitivity [52]. As such, these butyrate alterations in fibromyalgia warrant further investigation.

It is unclear if the elevated urinary lactate reported by Malatji study [53] was the L-isomer (which is endogenously produced in muscle metabolism), the D-form, which is primarily from exogenous (bacterial) sources [54] or both. D-lactic acidosis is a serious condition, associated with metabolic acidosis and encephalopathy. While no evidence exists that sub-clinical levels of D-lactate may contribute to other conditions, Rao et al. [55] maintain that mild cognitive symptoms produced by oral administration of glucose are attributable to the intestinal production of D-lactate. Dyscognition is a common symptom in fibromyalgia [56] and Malatji’s group did not report correlations in this domain with lactate.

The authors predicted succinate to be human in origin, as it is a metabolite of the Krebs Cycle [24]. However, gut bacteria also produce succinate from fibre, which is subsequently metabolised to propionate [57]. How the origin of succinate was determined to be human is unclear.

A notable strength of the work by Malatji et al., is the range of controls, including family members and cohabitants, allowing the researchers to consider shared factors that may influence results. However, scant information on drug use was provided in their reports [24, 25], nor was this controlled for, which is a significant limitation.

The overall diversity of gut microbiota, various influences on it and the resultant metabolites within any one population group present significant research challenges. The per os intake of any substrate or metabolite may increase populations of any organism able to consume it [42], and as such, measures of metabolites only give part of the picture. The use of both metagenomics and metatranscriptonomics in future studies may illuminate this issue.

### Other biomarkers

#### Intestinal Hyperpermeability

Goebel et al’s findings suggesting increased IP in both fibromyalgia and CRPS should be interpreted with caution [19]. The study protocol permitted participants to take non-steroidal anti-inflammatory drugs (NSAIDs) up to 48 h before and aspirin until commencing the study. Use of both these agents is associated with increased IP [58, 59]. Accordingly, higher levels of IP could reasonably be expected to occur secondary to use of the medications, but neither actual drug intake relative to test commencement, nor other factors known to be associated with altered IP were reported. Furthermore, Goebel’s study solution contained 10 g lactulose, 5 g mannitol and 20 g sucrose, and as a solution containing a much lower dose of lactulose and mannitol (5 g and 1 g respectively) can arrive in the colon within 2 h [60], the reliability of prediction of small intestinal permeability within this time-frame requires further investigation. Notably absent was a discussion about diarrhoea reported in fibromyalgia patients following this test [19], which could be related to osmotic effects of the saccharide load, or fermentation of same by bacteria. While a recent systematic review found relationships between increased IP and several chronic conditions [61], the validity of sugar-recovery tests as measures of IP and the applicability of increased IP to aberrations in human health beyond CD and inflammatory bowel diseases remains uncertain [62].

We commented previously on succinate, one of the metabolites contributing to differentiation of fibromyalgics and healthy controls, reported by Malatji (2017) [24]. Additionally, elevated circulating succinate [24] has been reported in patients with SIBO [63], IBS [64] *H. pylori* infection [65] and is associated with increased IP [66], indicating a gut-mediated inflammatory process. Whether any of these connections can be made for fibromyalgia patients with increased IP requires investigation.

#### Small intestinal bacterial overgrowth

The association between a positive hydrogen breath test and fibromyalgia [17] (Table 5), should be interpreted within the context of the limitations of the test, testing procedures and diagnostic criteria used. The authors regarded a normal breath test as having no rise in hydrogen or methane before 90 min of administration of lactulose, with “*a definitive rise never more than 20 ppm*” over 3 h of testing. Accordingly, most subjects would be expected to meet SIBO criteria, as lactulose is expected to initiate fermentation on arrival in the colon, which typically occurs within 100 mins [67]. Current criteria specify a diagnostic rise should occur before 90 min [68]. While hydrogen and methane were both measured in Pimentel’s 2004 study [17], test data was not tabulated and only hydrogen results were presented and discussed.

An association between fibromyalgia and SIBO was reported by Pimentel’s group in 2001 [69], a paper not included in our review due to unclear criteria for the diagnosis of both fibromyalgia and SIBO.

Fibromyalgics present with constipation at up to double the rate of those with diarrhoea [11, 70], which correlates with severity of fibromyalgia symptoms [70]. Constipation is associated with higher methane production [71], yet both Pimentel’s studies reported elevations in hydrogen gas [17, 69]. Despite the 2004 study being remarkable for its 100% detection rate of SIBO in fibromyalgics, to our knowledge no further studies have confirmed this relationship.

## Strengths & Limitations

A significant strength of this review is our requirement for clear and accepted criteria for the diagnosis of fibromyalgia. However, methodical differences, inconsistency in the testing methods used, inappropriate drug use and the inclusion of controls with diseases that were unaccounted pose limitations to our findings.

Oxidative stress markers, neurotransmitters or amino acids associated with pain signaling and neurological function were not included in our search terms. As alterations in several neurotransmitters, including serotonin and glutamate, have been implicated in fibromyalgia [7, 41] and bacteria may be involved in their synthesis [72], it could be argued that these terms should have been included. While some papers reporting these were picked up in our search, we determined that, as neurochemicals were not targeted in our search terms, the studies identified would not provide adequate substance for inclusion in a comprehensive review focusing on biomarkers associated with gastrointestinal dysfunction.

Despite these limitations and the heterogeneity of the studies included, preliminary findings suggest that gastrointestinal microbiome-targeted interventions may improve clinical and/or laboratory measures related to the symptoms of fibromyalgia.

## Conclusions

The findings of this review suggest the relationship between the gut microbiome and the pathophysiology of fibromyalgia is a largely underexplored area. Despite the limitations of the included studies, there are several indications that associations between the composition and metabolism of the gastrointestinal microbiota and fibromyalgia may exist. As such, well-designed studies that employ the latest technology and accepted diagnostic testing practices while accounting for confounding factors are warranted.

## Data Availability

Data sharing not applicable to this article as no datasets were generated. All datasets reviewed in this article are cited in the results section.

## Abbreviations

ACR: American College of Rheumatology
AUC: Area under the curve
CagA: Cytotoxin-associated gene A
CD: Coeliac disease
CNS: Central nervous system
CRPS: Chronic regional pain syndrome
FGID: Functional gastrointestinal disorder
FIQ: Fibromyalgia Impact Questionnaire
GABA: Gamma-Aminobutyric acid
IBS: Irritable bowel syndrome
IEL: Intraepithelial lymphocytosis
IgG: Immunoglobulin-G
IL: Interleukin
IP: Intestinal permeability
miRNA: micro ribonucleic acid
PRISMA: Preferred Reporting Items for Systematic Reviews and Meta-Analyses
qPCR: Quantitative polymerase chain reaction
SCFA: Short chain fatty acids
SIBO: Small intestinal bacterial overgrowth
RA: Rheumatoid arthritis
WPI: Widespread pain index
